# Intimate partner violence-related hospitalizations in Appalachia and the non-Appalachian United States

**DOI:** 10.1371/journal.pone.0184222

**Published:** 2017-09-08

**Authors:** Danielle M. Davidov, Stephen M. Davis, Motao Zhu, Tracie O. Afifi, Melissa Kimber, Abby L. Goldstein, Nicole Pitre, Kelly K. Gurka, Carol Stocks

**Affiliations:** 1 Department of Emergency Medicine and Social and Behavioral Sciences, West Virginia University, Morgantown, West Virginia, United States of America; 2 Department of Social and Behavioral Sciences, West Virginia University, Morgantown, West Virginia, United States of America; 3 Department of Epidemiology, West Virginia University, Morgantown, West Virginia, United States of America; 4 Departments of Community Health Sciences and Psychiatry, University of Manitoba, Winnipeg, Manitoba, Canada; 5 Department of Psychiatry and Behavioural Neurosciences, McMaster University, Hamilton, Ontario, Canada; 6 Department of Applied Psychology and Human Development, OISE, University of Toronto, Toronto, Ontario, Canada; 7 Faculty of Nursing, University of Alberta, Edmonton, Alberta, Canada; 8 Agency for Healthcare Research and Quality, Rockville, Maryland, United States of America; Indiana University, UNITED STATES

## Abstract

The highly rural region of Appalachia faces considerable socioeconomic disadvantage and health disparities that are recognized risk factors for intimate partner violence (IPV). The objective of this study was to estimate the rate of IPV-related hospitalizations in Appalachia and the non-Appalachian United States for 2007–2011 and compare hospitalizations in each region by clinical and sociodemographic factors. Data on IPV-related hospitalizations were extracted from the State Inpatient Databases, which are part of the Healthcare Cost and Utilization Project. Hospitalization day, year, in-hospital mortality, length of stay, average and total hospital charges, sex, age, payer, urban-rural location, income, diagnoses and procedures were compared between Appalachian and non-Appalachian counties. Poisson regression models were constructed to test differences in the rate of IPV-related hospitalizations between both regions. From 2007–2011, there were 7,385 hospitalizations related to IPV, with one-third (2,645) occurring in Appalachia. After adjusting for age and rurality, Appalachian counties had a 22% higher hospitalization rate than non-Appalachian counties (ARR = 1.22, 95% CI: 1.14–1.31). Appalachian residents may be at increased risk for IPV and associated conditions. Exploring disparities in healthcare utilization and costs associated with IPV in Appalachia is critical for the development of programs to effectively target the needs of this population.

## Introduction

Intimate partner violence (IPV) is a public health problem that involves victimization by a current or former spouse or partner through the use of physical and/or sexual violence, psychological harm, and in some cases, stalking [1]. Recent estimates demonstrate that approximately 37% of women and 31% of men in the United States (US) have reported experiences of IPV in their lifetime [2]. Although studies have revealed similar population rates of IPV between rural and non-rural locales [3],[4],[5], rural IPV is perpetrated at a higher frequency within relationships and with greater severity [6]. In rural areas, perpetrators of IPV are more likely to use weapons [7] and intimate partner homicide rates are significantly higher compared to those in urban and suburban locales [8]. Furthermore, those experiencing IPV in rural areas access medical systems and utilize formal and informal resources less frequently [9],[10]. Social and geographic isolation, increased travel times to receive shelter and treatment, and the presence of fewer social and medical support systems create significant challenges to the provision of adequate services for rural individuals exposed to IPV [5],[6],[11].

The culturally and geographically defined Appalachian region has one of the largest rural populations in the US (42% rural compared to 20% of US). Appalachia encompasses an area of about 205,000 square miles stretching along the spine of the Appalachian mountain range from southern New York to northern Mississippi [12]. Appalachian communities experience higher levels of economic distress characterized by lower income and educational attainment compared to those living in the non-Appalachian US [13]. Further, Appalachians have poorer health status, including higher rates of morbidity and mortality from chronic diseases, compared to individuals residing outside of the area [14],[15],[16]. The region also faces significant disparities related to mental health disorders and substance use [17] and higher death rates from prescription drug abuse [18] and motor vehicle crashes [19]. While these disparities are pronounced when compared to the rest of the US, there is substantial variation throughout Appalachia with central Appalachia continuing to face significant disparities compared to northern and southern Appalachia [14]. Appalachia also has a history of an extreme shortage of health care providers and appropriate health services [13],[20]. Therefore, examining social and contextual factors associated with health behaviors and outcomes among Appalachian populations, in specific, versus those with rural populations in general, is critical for the development of a comprehensive picture of localized Appalachian health disparities to guide the design and implementation of future interventions to reduce IPV.

Unfortunately, limited information is available regarding the extent and nature of IPV in Appalachia that is separate from what has been published about IPV in other rural regions. A recent population-based study of the prevalence of rural IPV conducted in 16 states found no significant difference in 12-month or lifetime IPV prevalence between those living in rural versus non-rural areas of the US, but only two of the included states have counties that lie within the Appalachian region [3]. Further investigation is warranted, as Appalachian populations—and in particular those residing in rural areas of Appalachia—may face double or triple disadvantage due to the intersection of geographical, sociocultural, and economic conditions unique to the region. These compounding levels of risk, coupled with the presence of fewer health resources [3],[4],[5], may leave this population inherently vulnerable to the acute and long-term physical and mental health consequences of IPV.

IPV-specific information is often captured from community, criminal justice, shelter, and healthcare settings. Although data from community samples allow for epidemiological study of the prevalence and incidence of various forms of IPV among the general US population or within specific communities, estimates are typically provided for single sites or at state or national levels. Data collected from criminal justice surveillance and shelter settings may include cases of IPV that involve law enforcement or criminal acts (eg, intimate partner homicide, use of weapons) and among populations who have left abusive or unsafe situations, respectively, but may not be generalizable to other situations involving IPV. Furthermore, these sources lack reliable data on the health impacts associated with IPV. Individuals exposed to IPV generally have more frequent contact with the healthcare system and higher costs for medical and mental health services than those who have not experienced IPV [21–24]. In fact, abused women are seen in the healthcare setting more often than in shelters or within the criminal justice system, making hospitalization records and surveillance data from health systems an important source of information on IPV.

Data on IPV-related hospitalizations, specifically, can provide a better picture of the demographics, injuries, comorbid conditions, and costs for individuals who have experienced the most serious forms of IPV [25],[26]. Only a few studies have examined characteristics associated with IPV-related hospitalizations, possibly due to limitations of available data, including incomplete medical record data, misclassification of IPV as other forms of trauma or accidents (possibly as a result of patients not disclosing abuse), and underuse of IPV-specific billing codes, which may result in insufficient sample sizes that preclude generation of reliable estimates of hospitalizations involving IPV. Rudman and Davey [27] utilized 1994 Healthcare Cost and Utilization Project (HCUP) data to examine the incidence of hospitalizations related to IPV for the entire US and reported that non-white and younger individuals were more likely to be hospitalized for IPV and have primary diagnoses related to acute injuries from violence, chronic disease, and mental health issues. Kernic and colleagues found that women who experienced IPV had an increased relative risk of hospitalization for assault, mental health issues, digestive system diseases, injuries and poisonings, and suicide attempts [28]. Statewide surveillance of inpatient discharge data and single site medical record reviews have also contributed to our knowledge surrounding inpatient healthcare utilization patterns associated with IPV [29],[30]. One study of two Level I trauma centers in Kalamazoo County Michigan found IPV-exposed individuals were ten times more likely to be hospitalized for injuries compared to national age-matched controls, and over half of the cases involved drugs and alcohol [29].

Although these few studies have enhanced our understanding of IPV-related hospitalizations, very little is known about inpatient care provided to individuals exposed to IPV residing in rural areas and no information is currently available regarding healthcare utilization and costs associated with IPV in the highly rural Appalachian region, despite the presence of multiple vulnerabilities that increase the risk for experiencing severe forms of IPV and associated health consequences [5]. Current research in this area involves sample sizes too small to make inferences about the Appalachian region as a whole or utilizes state or national level data that precludes county-level analyses required for examining the entirety of Appalachia. Data from the Healthcare Cost and Utilization Project, managed by the Agency for Healthcare Research and Quality, provide an opportunity to examine county-level hospitalization events, and patterns of healthcare utilization and costs in Appalachia. The objective of this study was to compare county-level population rates of IPV-related hospitalizations across Appalachia and non-Appalachian US counties, and to differentiate sociodemographic and clinical characteristics of the hospitalizations between the geographic areas.

## Methods

This study uses 2007–2011 data from the State Inpatient Databases, which are part of Healthcare Cost and Utilization Project. The Healthcare Cost and Utilization Project is a federal-state-industry partnership sponsored by the Agency for Healthcare Research and Quality that compiles and provides health data for healthcare policy and outcomes research [31]. The State Inpatient Databases’ files contain all inpatient records from community hospitals in each participating state. Collectively, these files contain clinical and non-clinical data on approximately 97% of all hospital discharges in the US. These data are standardized to permit multi-state and geographical comparisons [32]. IPV-related hospitalizations in Appalachian and Non-Appalachian counties were identified and extracted from the 2007–2011 intramural State Inpatient Databases’ files maintained by the Agency for Healthcare Research and Quality according to the methods detailed below. We received approval from the West Virginia University Institutional Review Board to carry out this study.

### Measures

#### Intimate partner violence hospitalizations

Our selection of codes to denote IPV-related hospitalizations within the State Inpatient Databases was guided by previous research on this topic [30],[33]. Specifically, we utilized the International Classification of Diseases, Ninth Revision, Clinical Modification (ICD-9-CM) codes for the following diagnoses: abuse by spouse/partner; adult maltreatment, unspecified; adult physical abuse; adult emotional/psychological abuse; adult sexual abuse; adult neglect–nutritional; other adult abuse and neglect; observation for abuse and neglect (S1 Table). A hospitalization was considered to be IPV-related if any of these codes were listed as a primary or secondary diagnosis. Primary diagnoses are those that are deemed chiefly responsible for the patient’s hospital admission while secondary diagnoses are all conditions that co-exist at the time of admission. Some records may have included codes used for other types of maltreatment or abuse that might not be considered IPV (eg, elder abuse, sexual assault outside of an intimate relationship). However, although research has demonstrated that codes specifying the perpetrator of abuse (eg, 9673 –abuse by spouse/partner) yield a high positive predictive value in terms of identifying true cases of IPV, they are used infrequently [30]. This presents a challenge of needing to balance increasing sensitivity at the expense of including false positives. Schafer *et al* found value in utilizing a “provisional” set of codes that are not directly indicative of IPV but indeed may be used in cases where IPV is present. While they span a broader definition than what is typically used to describe instances of IPV, they have been shown to have positive predictive values ranging from 40–97.6%. Thus, we opted to maximize the sensitivity of identifying IPV-related hospitalizations, recognizing that this may increase the possibility of capturing hospitalizations that might not be related to IPV.

#### Classification of Appalachian and non-Appalachian counties

The Appalachian Regional Commission is a federal, state, and local government partnership that was established by an act of Congress in 1965. In addition to promoting regional economic development, the Appalachian Regional Commission creates maps and conducts research on factors that affect economic development in the Appalachian region and provides a listing of Federal Information Processing Standard codes—five digit codes that uniquely identify counties and county equivalents in the US—to designate Appalachian counties [34]. There are 420 counties and eight independent cities that are considered “Appalachian” according to the Commission’s definition. Alabama does not contribute data to the State Inpatient Databases, therefore the 37 Appalachian counties in Alabama (approximately 9% of Appalachia as defined by the Commission) were excluded from the analysis, and thus the remaining 391 Federal Information Processing Standard codes were used to identify Appalachian Counties in the State Inpatient Databases. Data restrictions set forth by the Agency for Healthcare Research and Quality precluded our ability to compare Appalachian counties with all remaining (non-Appalachian) counties in the US, therefore we used simple random sampling to select 391 non-Appalachian counties as a comparison group. North Dakota’s 53 counties were excluded from the random sampling procedure because data from North Dakota were not available during two years of the five year study period.

#### Sociodemographic characteristics

We examined the following sociodemographic variables: age, sex, race, urban/rural location of patient residence, community income and primary payer. Patient age was measured in years. Sex was coded as *male* or *female*. Racial categories in the State Inpatient Databases include *White*, *Black*, *Hispanic*, *Asian/Pacific Islander*, *Native American*, and *Other*. Due to small sample sizes, we grouped *Asian/Pacific Islander* and *Native American* into the *Other* category. Patient residence was measured using the 2006 six-category urban-rural classification scheme for US counties developed by the National Center for Health Statistics. *Large central metropolitan* areas are “central” counties of metropolitan areas with ≥1 million population; *large fringe metro* areas are “fringe” counties of metro areas with ≥1 million population; *medium metro areas* are counties in metropolitan areas with 250,000 to 999,999 population; *small metro areas* are counties in metropolitan areas of 50,000 to 249,999 population; *micropolitan* areas are non-metropolitan counties with ≥10,000 population but less than 49,999; *non-core* areas are non-metropolitan and non-micropolitan counties. Community income was measured using the estimated median household income quartile for the patient’s zip code (*1st quartile* = ≤ $38,999; 2nd quartile = $39,000-$47,999; *3rd quartile* $48,000-$63,999; *4th quartile* ≥ $64,000). The expected primary payers included the following categories: *Medicare*, *Medicaid*, *private insurance*, and *Other* (includes Worker's Compensation, Civilian Health and Medical Program of the Uniformed Services [CHAMPUS], Civilian Health and Medical Program of the Department of Veterans Affairs [CHAMPVA], Title V, and other government programs).

#### Hospitalization characteristics

Variables related to hospital stay included admission day (*weekday* vs. *weekend*), year of hospitalization (calendar year), average length of hospital stay (days), in-hospital mortality (*yes* vs. *no*), hospital charges, comorbid diagnoses and procedures. Hospital charges (per hospitalization average and total) were measured in US dollars and represent the amount the hospital charged for the entire hospital stay, not including professional (physician) fees. The most commonly diagnosed conditions listed and procedures performed during the hospital stay were examined using the Agency for Healthcare Research and Quality’s Clinical Classification Software [35], which clusters thousands of ICD-9-CM diagnosis and procedures codes into a smaller number of meaningful categories.

#### Statistical analysis

Contingency table analyses were used to denote differences between Appalachian and non-Appalachian counties for the following variables: sex, race, urban/rural location of patient residence, community income, primary payer, admission day, year of hospitalization, in-hospital mortality, and discharge diagnoses and procedures performed during hospitalization. Differences in the average age, length of hospital stay, and hospital charge between Appalachian and non-Appalachian counties were tested using the *t*-test. To calculate IPV-related hospitalization rates, population counts by county and age group (<18, 18–34, 35–64, 65+) for the years 2007–2011 were extracted for all US counties from the US Census [36], excluding Alabama and North Dakota counties. From this final census file, we extracted the 391 non-Appalachian counties identified in our random sampling procedure to compare with data from the 391 Appalachian counties (excluding counties in Alabama and North Dakota). To control for the rurality of a county, 2013 Urban Influence Codes were recoded with codes ≥ 3 indicating nonmetropolitan (“rural”) counties and codes less than 3 indicating metropolitan (“urban”) counties. Poisson regression models were constructed to test differences in the rate of IPV-related hospitalizations between Appalachian and non-Appalachian counties. Negative binominal models were constructed if there was evidence of overdispersion or if the Lagrange multiplier test indicated that the negative binomial model was a better fit. A two-tailed alpha of 0.05 was selected as the threshold of statistical significance. All analyses were conducted using SAS 9.4 (SAS Institute).

## Results

Between 2007 and 2011, there were 7,385 IPV-related hospitalizations (2,645 Appalachian, 4,740 non-Appalachian). No statistically significant differences were found between the Appalachian and non-Appalachian counties with regard to sex, hospitalization admission day, length of stay, and in-hospital mortality (Table 1). Appalachian patients hospitalized for IPV were slightly older than those in non-Appalachian counties (53.4 years [range = 18–107] vs. 52.3 years [range = 18–104]; *p* = 0.04). Compared to the non-Appalachian region, individuals hospitalized in Appalachia were more likely to identify as White whereas hospitalized individuals in the non-Appalachian region were more likely to identify as Black or Hispanic. Almost two-thirds of patients hospitalized for IPV in Appalachia lived in communities with the lowest annual median income quartile (≤$38,999) compared with 35% of patients in non-Appalachian counties. Almost 15% of non-Appalachian IPV-related hospitalizations involved patients from neighborhoods in the wealthiest income quartile (>$64,000/year) versus 3% of Appalachian hospitalizations. A greater proportion of Appalachian patients utilized Medicare or Medicaid as their primary payer, while those in non-Appalachian counties were more likely to pay for their healthcare through private insurance.

**Table 1:** Sociodemographic variables and hospitalization characteristics^a^.

	Appalachian	Non-Appalachian	
Variable	(n = 2,645)	(n = 4,740)	p-value
	n (%)	n (%)	
**Age in years, Mean (SD)**	53.4 (21.1)	52.3 (21.7)	0.04
**Sex**			
Male	408 (15.4)	737 (15.6)	
Female	2237 (84.6)	4003 (84.5)	0.89
**Race**^**b**^			
White	1713 (88.9)	2508 (60.9)	
Black	162 (8.4)	815 (19.8)	
Hispanic	26 (1.5)	469 (11.4)	
Other^c^	27 (1.4)	326 (7.9)	
Missing	717	621	<0.001
**Location of patient residence**			
Large Central Metro	115 (4.4)	2326 (49.1)	
Large Fringe Metro	210 (7.9)	865 (18.3)	
Medium Metro	768 (29.0)	639 (13.5)	
Small Metro	455 (17.2)	275 (5.8)	
Micropolitan	610 (23.1)	396 (8.4)	
Non-core	487 (18.4)	239 (5.0)	<0.001
**Median income for patient zip code**^**d**^			
First quartile (≤$38,999)	1550 (60.6)	1594 (35.0)	
Second quartile ($39,000-$47,999)	679 (26.6)	1328 (29.1)	
Third quartile ($48,000-$63,999)	252 (9.9)	968 (21.2)	
Fourth quartile (≥$64,000)	76 (3.0)	668 (14.7)	
Missing	88	182	<0.001
**Primary Payer**			
Medicare	1211 (46.2)	1899 (40.2)	
Medicaid	756 (28.8)	1224 (25.9)	
Private insurance	350 (13.3)	807 (17.1)	
Self-pay	230 (8.8)	488 (10.3)	
No charge	13 (0.5)	36 (0.8)	
Other	63 (2.4)	273 (5.8)	<0.001
**Admission Day**			
Monday–Friday	1990 (75.2)	3492 (73.7)	
Saturday–Sunday	655 (24.8)	1248 (26.3)	0.14
**Year of Hospitalization**			
2007	317 (12.0)	828 (17.5)	
2008	710 (26.8)	940 (19.8)	
2009	575 (21.7)	889 (18.8)	
2010	495 (18.7)	1056 (22.3)	
2011	548 (20.7)	1027 (21.7)	<0.001
**In-hospital mortality**			
Yes	65 (2.5)	104 (2.2)	
No	2579 (97.5)	4625 (97.8)	0.48
Missing	1	11	
**Length of stay in days, Mean (SD)**	6.2 (10.5)	6.5 (12.2)	0.32
**Hospital charge, Mean**	$19,866	$30,995	< .0001

^a^Numbers may not add to 100% due to rounding.

^b^Some states in the State Inpatient Databases do not report race/ethnicity of discharges.

^c^*Other* includes *Asian/Pacific Islander*, *Native American*, *Other race*.

^d^Estimated median annual household income of residents in the patient's ZIP Code.

### Hospitalization rates

The IPV-related hospitalization rate was 14% higher in Appalachian counties compared to non-Appalachian counties (3.09 per 100,000 versus 2.71 per 100,000, respectively). After adjustment for age and rurality, Appalachian counties had a 22% higher rate of hospitalization related to IPV compared to non-Appalachian counties (RR: 1.22, 95% CI: 1.14–1.31) (Table 2).

**Table 2:** IPV-related hospitalizations for Appalachia and non-Appalachian counties in the US, 2007–2011.

	Appalachia		Non-Appalachian US			
	Number	Rate/100,000	Number	Rate/100,000	Rate Ratio	Adjusted Rate Ratio
					(95% CI)	(95% CI)
**Total**	2,645	3.09	4,740	2.71	1.14 (1.09–1.20)	1.22 (1.14–1.31)
**Age**						
18–34	604	2.56	1237	2.24	1.14 (1.04–1.26)	1.24 (1.08–1.41)
35–64	1147	2.53	1993	2.20	1.15 (1.07–1.24)	1.19 (1.08–1.32)
65+	894	5.34	1510	5.18	1.03 (0.95–1.12)	1.23 (1.10–1.37)
**Rurality†**						
Rural	1,026	3.27	565	2.60	1.26 (1.13–1.39)	1.26 (1.12–1.41)±
Urban	1,619	2.98	4,175	2.72	1.09 (1.03–1.16)	1.17 (1.08–1.26)

†Based on Urban Influence Codes.

±Poisson regression with Pearson scaling factor.

The top 15 most frequent diagnoses and procedures associated with IPV-related hospitalizations stratified by Appalachian and non-Appalachian counties are found in Tables 3 and 4. Mood disorders were a top diagnosis in both groups, but were twice as prevalent in Appalachia (20.2% vs. 10.0%). Substance use disorders and poisonings, urinary tract infections and pregnancy-related issues were also observed in both Appalachian and non-Appalachian counties; however, intracranial and internal injuries were among the top 15 diagnoses in non-Appalachian counties, only. Alcohol and drug rehabilitation/detoxification was the most common primary procedure indicated in both Appalachian and non-Appalachian regions, but occurred at a higher frequency within Appalachia (16.5% vs. 7.2%). Intubation, ventilation and blood transfusions were also common procedures performed for patients admitted to the hospital for IPV. Pregnancy-related procedures (delivery assistance, Cesarean section, fetal monitoring), psychiatric and psychological evaluation/therapy and treatment for wounds and fractures were also reported for patients in both regions.

**Table 3:** Top fifteen comorbid diagnoses for IPV-related hospitalizations in Appalachia and non-Appalachian counties in the US, 2007–2011.

Rank	Appalachia		Non-Appalachian US	
	Diagnosis category	n (%)	Diagnosis category	n (%)
1	Mood Disorders	533 (20.2)	Other injuries and conditions due to external causes	616 (13.0)
2	Other injuries and conditions due to external causes	233 (8.4)	Mood Disorders	476 (10.0)
3	Septicemia (except in labor)	88 (3.3)	Alcohol-related disorders	174 (3.7)
4	Urinary tract infections	81 (3.1)	Other complications of pregnancy	168 (3.6)
5	Fluid and electrolyte disorders	69 (2.6)	Septicemia (except in labor)	155 (3.7)
6	Schizophrenia and other psychotic disorders	67 (2.5)	Urinary tract infections	149 (3.4)
7	Poisoning by other medications and drugs	63 (2.4)	Intracranial injury	114 (2.4)
8	Pneumonia (except that caused by tuberculosis or sexually transmitted disease)	58 (2.2)	Poisoning by other medications and drugs	111 (2.3)
9	Poisoning by psychotropic agents	56 (2.2)	Fluid and electrolyte disorders	98 (2.1)
10	Chronic obstructive pulmonary disease and bronchiectasis	53 (2.0)	Acute and unspecified renal failure	97 (2.1)
11	Substance-related disorders	51 (1.9)	Schizophrenia and other psychotic disorders	92 (1.9)
12	Delirium, dementia, and amnestic and other cognitive disorders	50 (1.9)	Poisoning by psychotropic agents	85 (1.8)
13	Alcohol-related disorders	44 (1.7)	Crushing injury or internal injury	80 (1.7)
14	Diabetes mellitus with complications	42 (1.6)	Diabetes mellitus with complications	77 (1.6)
15	Other complications of pregnancy	42 (1.6)	Delirium, dementia, and amnestic and other cognitive disorders	68 (1.4)

**Table 4:** Top fifteen primary procedures for IPV-related hospitalizations in Appalachia and non-Appalachian counties in the US, 2007–2011.

Rank	Appalachia†		Non-Appalachian US‡	
	Procedure category	n (%)	Procedure category	n (%)
1	Alcohol and drug rehabilitation/detoxification	169 (16.5)	Alcohol and drug rehabilitation/detoxification	152 (7.2)
2	Respiratory intubation and mechanical ventilation	82 (8.0)	Respiratory intubation and mechanical ventilation	133 (6.3)
3	Blood transfusion	61 (6.0)	Blood transfusion	97 (4.6)
4	Other vascular catheterization; not heart	54 (5.3)	Other vascular catheterization; not heart	94 (4.5)
5	Upper gastrointestinal endoscopy; biopsy	34 (3.3)	Other therapeutic procedures	80 (3.8)
6	Debridement of wound; infection or burn	33 (3.2)	Other procedures to assist delivery	74 (3.5)
7	Indwelling catheter	32 (3.1)	Suture of skin and subcutaneous tissue	67 (3.2)
8	Other procedures to assist delivery	32 (3.1)	Upper gastrointestinal endoscopy; biopsy	63 (3.0)
9	Psychological and psychiatric evaluation and therapy	28 (2.7)	Debridement of wound; infection or burn	58 (2.8)
10	Cesarean section	26 (2.5)	Psychological and psychiatric evaluation and therapy	54 (2.8)
11	Incision of pleura; thoracentesis; chest drainage	23 (2.3)	Computerized axial tomography (CT) scan head	53 (2.5)
12	Treatment; facial fracture or dislocation	18 (1.8)	Cesarean section	47 (2.2)
13	Other therapeutic procedures	18 (1.8)	Hemodialysis	42 (2.0)
14	Hemodialysis	17 (1.7)	Fetal monitoring	35 (1.7)
15	Other non-OR therapeutic procedures on skin and breast	17 (1.7)	Treatment; facial fracture or dislocation	33 (1.6)

† Out of 1,023 hospitalizations in which procedures were performed.

‡ Out of 2,098 hospitalizations in which procedures were performed.

## Discussion

Socioeconomically disadvantaged and rural communities are considered health disparity groups by the National Institutes of Health [36]. Some areas within the largely rural region of Appalachia face considerable disadvantage and disparities related to mental health, chronic disease, substance abuse and injury and available preventive services and treatment for these conditions [14–20]. The current study adds to the literature by providing new information about healthcare utilization and costs associated with IPV in Appalachia. To the best of our knowledge, this is the first study to examine IPV-related hospitalizations in Appalachia and make comparisons with non-Appalachian counties. To successfully complete this analysis, it was necessary to use the restricted, intramural State Inpatient Databases’ files at the Agency for Healthcare Research and Quality due to variable restrictions on the publicly available data files.

Our primary finding was that Appalachian counties had a significantly higher rate of IPV-related hospitalizations compared to a sample of non-Appalachian counties. The explanation for the difference between the two regions is likely multifactorial, though characteristics associated with rurality may partially account for the findings. While previous research has shown rates of IPV to be roughly equivalent in urban and rural areas [5], some studies have demonstrated individuals living in inner-city urban areas to be at increased risk for IPV and IPV-related health consequences [4],[37–40]. While Appalachia is twice as rural as the non-Appalachian US, over half of the region is comprised of suburban or urban areas. In the current study, the highest rates of IPV-related hospitalizations were found in rural Appalachian counties. Thus, it is plausible that rural areas within Appalachia are inherently different from the suburban and urban locales within the region (non-rural Appalachia) as well as other rural, non-Appalachian areas of the US. This highlights the importance of examining the urban-rural continuum regionally versus solely at state or national levels. Edwards’ recent critical review of the IPV literature found rural IPV to be more chronic and severe and result in worse physical and psychosocial health outcomes for individuals who have experienced violence [5], and Logan and colleagues found that perpetrators of IPV in rural areas are more likely to use knives or guns—injuries which may necessitate hospitalization [7]. Additionally, rural locales have fewer IPV services and IPV-exposed individuals in rural areas report less help seeking behaviors and more difficulty accessing medical care [5]. Further, compared with the rest of the US, Appalachian counties report higher aggregate healthcare costs and more disparities in access to care [41]. Thus, the higher IPV-related hospitalization rate in Appalachia might not be a function of a higher prevalence of IPV, but instead may reflect overutilization of emergency or inpatient services due to shortages in preventive and IPV-specific services in the medically underserved region. Because IPV frequency and severity increases over time, individuals without adequate means to address IPV might remain in situations of escalating abuse that eventually require emergent inpatient care. This disparity in access to resources may be especially pronounced among Appalachian residents living in the most rural, isolated areas.

In addition to rurality, sociodemographic differences between the two regions may explain Appalachia’s higher rate of IPV-related hospitalizations. Appalachian patients were older than their non-Appalachian counterparts, were more likely to identify as White, and have public versus private insurance. They were also more likely to reside in the poorest neighborhoods—only 3% of Appalachian patients hospitalized for IPV lived in communities with the highest income quartile of >$64,000 per year. These data are reflective of the Appalachian population as a whole: residents of Appalachia are more racially homogenous (83% White) than the total US population (63% White), significantly older (median age = 40 years vs. 37 years), and have a higher proportion of residents aged 65 years and older (16% vs 13%). Appalachians are also less likely to have at least a bachelor’s level of education (22% vs. 29%) and they have significantly lower incomes than non-Appalachian residents. Moreover, the households with the lowest incomes in Appalachia are found within the most rural counties [42]. In a recent study, Edwards *et al* [43] found that rural communities with high poverty and low collective efficacy (ie, social cohesion among community members and willingness to intervene for the common good) [44] experienced the highest rates of IPV. Low income levels in particular have been shown to be one of the strongest predictors of IPV, even after controlling for race/ethnicity [45]. In addition, while many studies have found racial/ethnic minority populations disproportionately experience IPV [40],[46–48] and are more likely to be hospitalized as a result [28], less is known about IPV risk and outcomes among White populations living in poor, predominately White communities [49]. While large amounts of missing data on race/ethnicity within the State Inpatient Databases precluded our ability to reliably control for race/ethnicity in our regression analyses, examining IPV risk, healthcare utilization patterns, and health and psychosocial outcomes across racial/ethnic groups in Appalachia is an important next step for future research.

While it is not surprising that Appalachian patients were slightly older than non-Appalachian counterparts, the older age of patients in both regions contrasts with other studies that have found younger (≤30) populations are at increased risk for IPV-related hospitalizations [27]. However, Shafer *et al* reported that although most patients exposed to IPV who sought emergency department services were between ages 20–29, those 50 years or older were more frequently hospitalized [30]. This suggests that, while younger groups may be at increased risk for IPV, older patients who have experienced IPV may be more likely to be hospitalized due to comorbidities and other chronic conditions (eg, osteoporosis that contributes to more severe fractures, anticoagulants that contribute to more extensive bleeding).

The top diagnoses shown in Table 3 mirror what has been reported in other studies of IPV-related hospitalizations, wherein mental disorders, suicide attempts, traumatic injuries and assault, drug addiction and poisonings, and pregnancy complications were listed as top diagnoses among hospitalized patients [27],[28],[30]. There were notable differences in comorbid diagnoses and procedures between Appalachian and non-Appalachian patients, however. Comorbidities related to substance use and mental health issues were more frequently observed among Appalachian patients while non-Appalachian patients had more diagnoses and procedures indicative of acute IPV-related injuries (eg, intracranial, crushing, and internal injuries, sutures, CT scans). The high prevalence of comorbid diagnoses and procedures related to both mental health and substance use disorders among Appalachian patients with hospitalizations related to IPV is noteworthy, but not unexpected, as the co-occurrence of IPV, mental health issues and substance use is well-established [24],[50–53]. Likewise, Appalachia faces significant disparities related to mental health and substance use disorders, including epidemic rates of prescription opioid and heroin use. Using state, sub-state, and county level data from 2000 to 2005, Zhang found that Appalachian adults have a higher prevalence of mental health disorders compared to the rest of the nation, specifically with regard to psychological distress and major depressive disorder and that use rates of tobacco, prescription opiates, and psychotherapeutics (ie, pain relievers, tranquilizers, stimulants, sedatives) are higher in Appalachia compared with the US, especially in coal mining communities [17]. Furthermore, while Appalachians are admitted to treatment for the use of opiates and synthetic drugs at a higher rate than in other regions of the US, there are fewer substance abuse treatment facilities that offer outpatient detoxification in Appalachia when compared to facilities outside of the region and more patients in Appalachia are admitted to community hospitals for mental health or substance abuse treatment via the emergency department, especially in coal mining areas and counties with the greatest economic distress [17]. Together, these data may explain why, in the current study, ‘Alcohol and drug rehabilitation/detoxification’ was the top procedure code listed among patients with IPV-related hospitalizations in both regions, but was observed much more frequently in Appalachian counties.

Urinary tract infections and pregnancy-related issues were prevalent in Appalachian and non-Appalachian groups. Previous research has found women experiencing IPV are more likely to be diagnosed with urinary tract infections [54],[55] and pregnancy may increase IPV risk [56],[57], especially among women with lower socioeconomic status [58]. Lipsky and colleagues found women reporting IPV exposure were more likely to be hospitalized during pregnancy with diagnoses related to injuries, violence, pregnancy complications, substance abuse and mental health issues [59].

### Implications

#### Research

The higher rate of IPV-related hospitalizations in Appalachia, coupled with the greater proportion of hospitalizations for patients with comorbid mental health and substance use disorder diagnoses, underscores the need to study the complex interplay between IPV, mental health issues, and substance use among individuals living in Appalachia. In particular, more research is needed to explore the relationship between macro-level (e.g., social, economic, cultural) and individual (e.g., personal, behavioral, physiological) factors, IPV rates and medical care/healthcare utilization in Appalachian communities.

Because these data may represent the most severe forms of IPV that required hospitalization, it is likely that such hospitalizations are only the tip of the epidemiologic iceberg [60] for the problem of IPV in Appalachia. However, it is difficult to obtain accurate estimates of IPV and related hospitalizations in Appalachia using current data. National, statewide, and local surveillance data are often limited in detecting differences across geographic domains and in providing comprehensive information about high-risk subgroups [61]. Where possible, data sources from healthcare and other sectors should include county-level data to promote the calculation of more accurate IPV estimates across geographic regions. Furthermore, the lack of a gold standard for identifying IPV in healthcare settings and underuse of IPV-specific diagnosis codes (and perpetrator e-codes in particular) complicate valid and reliable examinations of IPV-related healthcare costs and utilization patterns. Researchers are often faced with the decision to examine a small number of codes with high specificity for identifying IPV, recognizing that this results in a vast underestimate of the issue due to underuse of IPV-specific diagnosis codes. Casting a wider net by examining a broader range of codes that are less specific, but more sensitive, in identifying true cases of IPV, increases the risk for capturing false positives. This trade-off is a noted limitation in IPV research, generally, and partially explains the significant variation in IPV estimates among different target populations and across different settings [33]. Uniform definitions and consistent coding for IPV among healthcare professionals, billers, and payers, are necessary to maximize the utility of using health systems data for IPV surveillance [30].

#### Policy

Our results point to the need for greater emphasis on prevention and intervention programs for IPV in Appalachia and other rural, underserved areas. Areas with limited health and social resources in particular may benefit from community collaboration and coordinated response to conserve resources, leverage local capabilities, and prevent the duplication of efforts to address IPV. Increased communication and cross-trainings for those involved with IPV in various sectors (eg, healthcare, criminal justice, policymakers, payers, advocacy), as well as increased resources and funding for these efforts is vital for comprehensive service provision.

#### Practice

Clinically, these findings highlight the complex nature of treating IPV and associated consequences in a healthcare system already facing resource shortages and time constraints. Screening for IPV has been recommended for patients presenting to healthcare settings [62], especially for those admitted to the hospital with traumatic injuries [63], but linkages with appropriate IPV-related services are needed for screening to have a positive impact. Furthermore, due to the frequent co-occurrence of psychiatric and substance use conditions, hospitalized patients who have experienced IPV in Appalachia may require a higher level of post discharge care after returning home from the hospital, including access to substance use and mental health treatment resources. Integrated treatment strategies that address both physical and mental health conditions for patients hospitalized for IPV should be considered to prevent re-hospitalization and recurrence of IPV. Appalachian healthcare providers should be knowledgeable of available community resources including social work services to assist with case management and linkage to social services and substance abuse treatment and counseling. While this continuity of care is critical, it may be challenging given the limited resources present in many Appalachian communities. New initiatives such as establishing patient-centered medical homes that provide comprehensive care using diverse healthcare teams, telemedicine, and use of patient navigators to meet the needs of underserved patients hold significant promise for improving access to healthcare and ancillary services in rural Appalachia [64],[65]. These system-level transformations, if successful, will also increase the likelihood that individuals experiencing IPV will come into contact with the healthcare system, improving their chances to receive support and intervention.

### Limitations

The results of this study should be interpreted in light of several limitations. While we chose codes to characterize IPV-related hospitalizations based on previous research, these codes have the potential to capture abuse between adults that are not in intimate relationships (eg, “adult maltreatment not otherwise specified”). This might lead to non-IPV cases being included in our sample, resulting in an overestimation of hospitalizations related specifically to violence between intimate partners. However, this bias would be equally present in both Appalachian and non-Appalachian hospitalizations, given that we used the same codes for each region in our comparisons. Furthermore, it is likely that the true number of IPV-related hospitalizations is greater than what is reported here due to underutilization of billing codes related to violence and injury within medical record data. This underestimation may be exacerbated in Appalachian or rural counties, as these groups may be underrepresented in healthcare data [66]. Reluctance to disclose IPV due to social stigma and fear of increased perpetration may be exacerbated in rural areas with tight knit social networks [3],[5]. Furthermore, because Alabama is one of two states that does not contribute to the SID, we were not able to include data from the 27 Appalachian counties in Alabama, which accounts for 9% of Appalachia. North Dakota was also excluded from our sampling frame of non-Appalachian counties because they did not contribute at least two years of data during the study period. Nevertheless, to our knowledge, this is the most comprehensive examination of IPV in the Appalachian region. Additionally, the unit of analysis in the State Inpatient Databases is the individual hospitalization. Thus, it is possible that patients may be counted more than once if they have repeated hospitalizations within a calendar year. We were also not able to control for race/ethnicity due to missing data. Lastly, due to data limitations, we were unable to explore specific characteristics of IPV-related hospitalizations, especially whether the hospitalized patient was a victim or survivor of violence, a perpetrator, or if the violence was bidirectional. Therefore, given that perpetrators are also seen in inpatient settings [29], it is possible that some of our hospitalizations included patients other than those who were solely IPV victims or survivors.

## Conclusions

Hospitalizations for IPV were disproportionately higher in Appalachian counties in the US, suggesting a possible health disparity issue. Exploring potential disparities in healthcare utilization and costs associated with IPV in the Appalachian region is critical for the development of future interventions and prevention programs to effectively target the needs of this population. Other public health problems that disproportionately affect Appalachian communities (eg, cancer, obesity, prescription opioid abuse) have received federal and local attention and increased funding. Funding is needed for future research to examine the unique contextual factors that contribute to IPV in Appalachian communities, and the potential relationship between IPV and other health disparities in the region.

## Supporting information

S1 Table Table of diagnostic codes. This table contains the list of diagnostic codes used to identify IPV-related hospitalizations.
(DOCX)
