# Supplementary material for: Intimate partner violence-related hospitalizations in Appalachia and the non-Appalachian United States
Source: PLoS One. 2017 Sep 8;12(9):e0184222. doi: 10.1371/journal.pone.0184222 (PMC5590902; doi:10.1371/journal.pone.0184222)
Supplement: S1 Table — This table contains the list of diagnostic codes used to identify IPV-related hospitalizations. (DOCX) [file pone.0184222.s001.docx]

# Supporting Information S1 Table

## S1 Table. Diagnostic codes used to identify IPV-related hospitalizations

| ICD-9-CM Code* | Description |
| --- | --- |
| E9673 | abuse by spouse/partner |
| 99580 | adult maltreatment, unspecified |
| 99581 | adult physical abuse |
| 99582 | adult emotional/psychological abuse |
| 99583 | adult sexual abuse |
| 99584 | adult neglect – nutritional |
| 99585 | other adult abuse and neglect |
| V7181 | observation for abuse and neglect |
| * International Classification of Diseases, Ninth Revision, Clinical Modification | |
